# Shared genetic architecture between schizophrenia and suicide attempt in European populations: fine-mapping and functional annotation

**DOI:** 10.1192/j.eurpsy.2026.11272

**Published:** 2026-07-31

**Authors:** Y. Deng

**Affiliations:** King’s College London, London, United Kingdom

## Abstract

**Introduction:**

Individuals with schizophrenia (SCZ) have an increased risk of suicide attempt (SA) and early mortality. Although previous studies have reported a genetic correlation between the two traits, the underlying mechanisms remain unclear. This study aims to investigate the shared genetic basis of SCZ and SA, and to identify potential causal variants and biological pathways.

**Objectives:**

This study will (1) quantify the genetic architecture shared between SCZ and SA; (2) test whether the genetic overlap is better explained by a causal pathway or by pleiotropic effects; (3) identify genomic loci and causal variants that contribute to both traits; (4) characterize the biological pathways and brain regions enriched among the shared loci.

**Methods:**

European-ancestry GWAS summary statistics from UKB, PGC and FinnGen (93,257 cases and 1,908,506 controls) were meta-analysed for SCZ and SA, respectively. SNP heritability and cross-trait genetic correlation were estimated via LD score regression. Mendelian randomisation and CAUSE distinguished causal from pleiotropic effects. Cross-trait meta-analysis using ASSET identified loci with shared association signals, followed by fine-mapping with SuSiE and regional colocalisation (coloc). Functionally relevant genes were prioritised using FUMA and MAGMA.

**Results:**

The liability scale *h^2^* was 0.118 for SCZ and 0.046 for SA. The genetic correlation between SCZ and SA was significant (r_g_ = 0.42, SE = 0.03, p = 5.67e-54). MR analyses supported a positive causal effect of SCZ on SA (Table 1). However, CAUSE indicated that the causal model did not fit better than the sharing model significantly (p = .83). Fine-mapping prioritized 937 and 122 high-confidence credible sets (PIP > 0.5) across 192 and 28 risk loci for SCZ and SA, respectively. Among these, 20 loci contained credible sets in both traits, of which 6 showed strong evidence for shared causal variants (PP.H₄ > 0.9). These loci were mapped to genes including DRD2, ELAVL4, TSNARE1, which were also significant in gene-based test (Fig. 1). Tissue expression analysis showed no significant enrichment across GTEx tissues, although top signals were observed in cerebellum and cortical regions (Fig. 2). Gene-set analysis suggested significant enrichment of pathways related to synaptic structure and cellular stress regulation.

**Table 1.** MR estimates (SCZ → SA) Table 1. Long description.

Method	SNPs	β (SE/SD)	p	Cochran’s Q (p)	Egger intercept (p)
IVW	160	0.148 (0.023)	< .001	219.1 (159), = 0.001	
MR Egger	160	0.055 (0.123)		218.3 (158), = 0.001	0.006 (= 0.443)
**MR-PRESSO (Raw)**	160	0.148 (0.023)	1.378		
**MR-PRESSO (Corrected)**	153	0.156 (0.022)	9.100

**Image 1:**
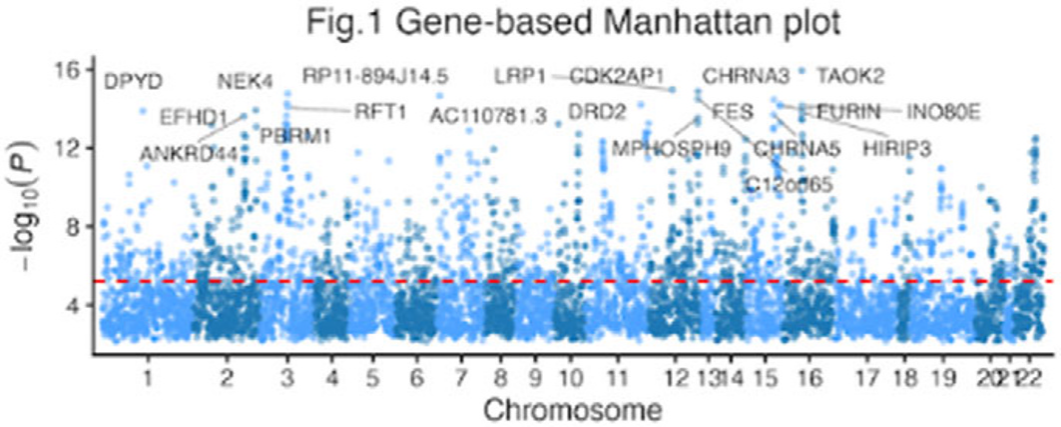
Image 1: Long description.

**Image 2:**
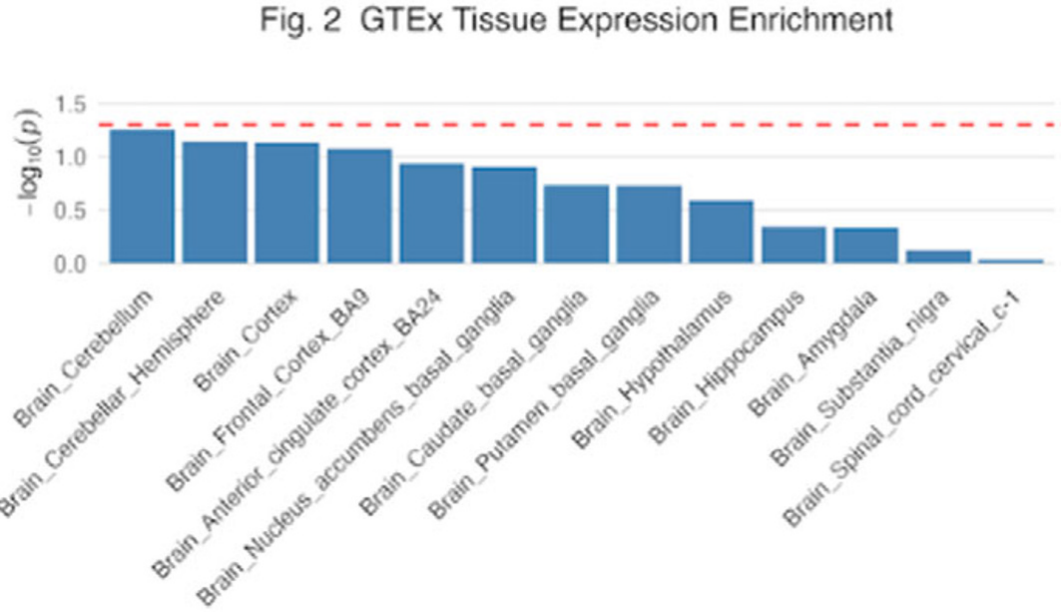
Image 2: Long description.

**Conclusions:**

Genetic overlap between SCZ and SA is more likely driven by pleiotropic effects involving synaptic and stress-related pathways, rather than a direct causal link. These findings may inform the prevention of suicide in people with SCZ.

**Disclosure of Interest:**

None Declared

## Long descriptions

### Table 1. Long description

The table consists of six columns: Method, S N P s, beta (S E / S D), p, Cochran’s Q (p), and Egger intercept (p).

* Row 1: I V W method with 160 S N P s, beta of 0.148 (0.023), p less than .001, and Cochran’s Q of 219.1 (159), = 0.001.

* Row 2: M R Egger method with 160 S N P s, beta of 0.055 (0.123), Cochran’s Q of 218.3 (158), = 0.001, and Egger intercept of 0.006 (= 0.443).

* Row 3: M R-P R E S S O (Raw) with 160 S N P s, beta of 0.148 (0.023), and p of 1.378.

* Row 4: M R-P R E S S O (Corrected) with 153 S N P s, beta of 0.156 (0.022), and p of 9.100.

Navigate back to Table 1..

### Image 1: Long description

The X-axis is labeled Chromosome and ranges from 1 to 22. The Y-axis is labeled minus log sub 10 (P) and ranges from 0 to 16. A horizontal red dashed line indicates the significance threshold at approximately Y equals 5. Data points are plotted as blue dots, alternating between light and dark blue for each chromosome.

Significant peaks above the threshold are labeled with gene names from left to right:

* Chromosome 1: D P Y D, E F H D 1, and A N K R D 44.

* Chromosome 2: N E K 4 and P B R M 1.

* Chromosome 3: R P 11 minus 894 J 14 dot 5 and R F T 1.

* Chromosome 7: A C 110781 dot 3.

* Chromosome 8: L R P 1.

* Chromosome 10: C D K 2 A P 1 and D R D 2.

* Chromosome 12: M P H O S P H 9 and F E S.

* Chromosome 14: C H R N A 3 and C 12 o r f 65.

* Chromosome 15: C H R N A 5 and F U R I N.

* Chromosome 16: T A O K 2.

* Chromosome 18: H I R I P 3.

* Chromosome 19: I N O 80 E.

The highest peak reaches a Y-value of approximately 16 on Chromosome 16, labeled T A O K 2.

Navigate back to Image 1:.

### Image 2: Long description

The Y-axis is labeled minus log sub 10 (p) with a scale from 0.0 to 1.5. A horizontal red dashed line indicates a significance threshold at approximately 1.3. The X-axis lists 13 brain regions. The blue bars show a downward trend from left to right.

* Brain_Cerebellum is the highest bar, nearly reaching the 1.3 threshold.

* Brain_Cerebellar_Hemisphere and Brain_Cortex follow at approximately 1.1.

* Brain_Frontal_Cortex_B A 9 is just above 1.0.

* Brain_Anterior_cingulate_cortex_B A 24 and Brain_Nucleus_accumbens_basal_ganglia are around 0.9.

* Brain_Caudate_basal_ganglia and Brain_Putamen_basal_ganglia are approximately 0.7.

* Brain_Hypothalamus is at 0.6.

* Brain_Hippocampus and Brain_Amygdala are approximately 0.3.

* Brain_Substantia_nigra is approximately 0.1.

* Brain_Spinal_cord_cervical_c-1 is the lowest, near 0.0.

Navigate back to Image 2:.

